# Transcriptomic analysis supports a role for the nervous system in regulating growth and development of *Fasciola hepatica* juveniles

**DOI:** 10.1371/journal.pntd.0010854

**Published:** 2022-11-07

**Authors:** Emily Robb, Erin M. McCammick, Duncan Wells, Paul McVeigh, Erica Gardiner, Rebecca Armstrong, Paul McCusker, Angela Mousley, Nathan Clarke, Nikki J. Marks, Aaron G. Maule

**Affiliations:** Microbes & Pathogen Biology, The Institute for Global Food Security, School of Biological Sciences, Queen’s University Belfast, Belfast, United Kingdom; Aberystwyth University - Penglais Campus: Aberystwyth University, UNITED KINGDOM

## Abstract

*Fasciola* spp. liver flukes have significant impacts in veterinary and human medicine. The absence of a vaccine and increasing anthelmintic resistance threaten sustainable control and underscore the need for novel flukicides. Functional genomic approaches underpinned by *in vitro* culture of juvenile *Fasciola hepatica* facilitate control target validation in the most pathogenic life stage. Comparative transcriptomics of *in vitro* and *in vivo* maintained 21 day old *F*. *hepatica* finds that 86% of genes are expressed at similar levels across maintenance treatments suggesting commonality in core biological functioning within these juveniles. Phenotypic comparisons revealed higher cell proliferation and growth rates in the *in vivo* juveniles compared to their *in vitro* counterparts. These phenotypic differences were consistent with the upregulation of neoblast-like stem cell and cell-cycle associated genes in *in vivo* maintained worms. The more rapid growth/development of *in vivo* juveniles was further evidenced by a switch in cathepsin protease expression profiles, dominated by cathepsin B in *in vitro* juveniles and by cathepsin L in *in vivo* juveniles. Coincident with more rapid growth/development was the marked downregulation of both classical and peptidergic neuronal signalling components in *in vivo* maintained juveniles, supporting a role for the nervous system in regulating liver fluke growth and development. Differences in the miRNA complements of *in vivo* and *in vitro* juveniles identified 31 differentially expressed miRNAs, including *fhe-let-7a-5p*, *fhe-mir-124-3p* and miRNAs predicted to target Wnt-signalling, which supports a key role for miRNAs in driving the growth/developmental differences in the *in vitro* and *in vivo* maintained juvenile liver fluke. Widespread differences in the expression of neuronal genes in juvenile fluke grown *in vitro* and *in vivo* expose significant interplay between neuronal signalling and the rate of growth/development, encouraging consideration of neuronal targets in efforts to dysregulate growth/development for parasite control.

## Author summary

Parasitic worms are notoriously difficult to study outside of a host organism. However, recent developments in culture methods for *Fasciola hepatica* liver fluke juveniles support growth and development of these parasites in the laboratory (*in vitro*) towards adult parasites. Having the ability to grow pathogenic juvenile stages *in vitro* enables functional studies to validate potential drug and vaccine targets. However, comparison of *in vitro* grown juveniles to juveniles retrieved from infected hosts (*in vivo*) shows considerable size differences suggesting at least some differences in biology that could undermine the relevance of data generated from *in vitro* maintained parasites. This study examines gene expression differences between *in vitro* and *in vivo* maintained *F*. *hepatica* juveniles via transcriptomic analysis to identify similarities and differences in their biology which may help explain differences in the rate of growth and development. 86% of genes were shown to be expressed at similar levels across treatment groups suggesting a high level of biological similarity between *in vitro* and *in vivo* juveniles. However, the genes that are expressed differently between these juveniles will help improve current culture methods and provide a new group of potential drug targets that impact on juvenile growth and development.

## Introduction

*Fasciola spp*. liver flukes are important helminth pathogens with far reaching global impacts on veterinary and human medicine causing a disease known as fasciolosis [1]. Global agricultural losses associated with *Fasciola* infection are estimated at around US$3.2 billion annually [2], although this is thought to be a considerable underestimation as *F*. *hepatica* parasites in particular are known to infect a wide range of mammalian hosts across broad geographical ranges [3,4]. Designated a neglected tropical disease by the World Health Organisation (WHO) [1] due to its impact on human populations, *F*. *hepatica* is thought to infect up to 17 million people, with a further 91 million people at risk of infection worldwide [5].

Treatment of the early pathogenic stages of fasciolosis relies on the drug Triclabendazole, since other flukicides do not kill young juveniles [4,6]. Definitive mammalian hosts are infected through the ingestion of infectious metacercariae encysted on vegetation [7,8]. Newly excysted juveniles (NEJs) emerge in the duodenum and migrate through the intestinal wall to the liver parenchyma, causing acute stages of disease associated with host blood loss and even death with high burden infections [7–9]. Although adults residing in the bile duct are reproductively active and associated with chronic infection, the major pathology associated with fasciolosis is caused by juvenile migration within the first few weeks of infection [10, 11]. The absence of a liver fluke vaccine and increasing anthelmintic resistance threaten the sustainability of liver fluke control. Triclabendazole treatment failure is widely reported for livestock and more recent cases of emerging resistance in human populations is deeply concerning and highlights the pressing need for new flukicides, particularly targeting the highly pathogenic early-stage juveniles [4,12–14].

Despite the pathology associated with early-stage infections, there is a dearth of information on the biology of migrating juveniles due to their small size, liver parenchyma location and, historically, the absence of readily amenable *in vitro* culture methods [15]. At this stage of development, juveniles are growing and moving rapidly through host tissues, encountering different microenvironments and host responses [11]. Understanding the biology of these behaviours and host-parasite interactions will support new target discovery and control option developments. Research into helminth infections often relies on the use of animal models to inform host-parasite relationships. However, such studies are often challenging, of variable relevance to livestock/human hosts and provide limited opportunity to interrogate parasite biology [16]. *In vitro* culture, where possible, supports curiosity driven research and provides many advantages to early-stage studies of parasite biology. Despite recent advances in propagating the full life cycle of *Schistosoma mansoni* [17], *in vitro* parasite culture is notoriously difficult and, where it is possible, the approaches need to reasonably replicate *in vivo* parasite biology such that readouts have relevance to control.

McCusker *et al*. [15] developed a chicken serum-based culture platform that promotes sustained *F*. *hepatica* juvenile survival, growth and development *in vitro*. This has facilitated the development of a robust functional genomics platform that promotes discovery-based studies that seed drug target identification and validation in the most damaging life stage [18]. Although extremely robust, the current culture platform is believed to support a slower rate of growth and development of *F*. *hepatica* juveniles *in vitro* when compared to *in vivo* counterparts and, whilst *in vitro* juveniles start to develop adult like features, they do not progress to egg laying adults [15]. In the absence of culture methods that allow full life cycle propagation, it is important to ascertain if the slower growing *in vitro* worms resemble their *in vivo* counterparts to ensure functional genomics studies on *in vitro* cultured juveniles have relevance to control.

Taking advantage of recent developments in genomic and transcriptomic resources for *F*. *hepatica*, we present in depth comparative transcriptomic analysis of 21 day *in vitro* maintained *F*. *hepatica* juveniles and stage matched 21 day *in vivo F*. *hepatica* juveniles, describing key differences in gene expression in the most pathogenic life stage under differing growth conditions. At this early stage *in vivo*, juveniles are actively migrating through liver parenchyma but have not yet developed reproductive organs, comparable with their *in vitro* counterparts. Although the expression of core biological functioning components is consistent in comparisons between the two growth conditions, the differences that are evident can mostly be explained by the disparate rates of growth and cell proliferation observed in the two groups. Notably, protease expression profiles differ and corroborate the hypothesis that time-matched *in vitro* maintained juveniles develop more slowly than *in vivo* juveniles, such that, the *in vitro* juveniles are at an earlier stage of development. The data indicate a key role for miRNAs in driving the developmental differences between *in vitro* and *in vivo* maintained juveniles with over one third of the identified miRNAs being differentially expressed. Further, profound differences in the expression of genes associated with neoblast proliferation and development are consistent with the observed higher levels of neoblast-like stem cell proliferation *in vivo*. The unexpected down-regulation of multiple nervous system associated signalling pathways *in vivo* exposes an important role for the nervous system of *F*. *hepatica* in the modulation of juvenile growth and development, encouraging efforts to identify neuronal signalling pathways that dampen cell proliferation and/or growth dynamics.

## Materials and methods

### Ethics statement

This work was carried out in accordance with the Animals (Scientific Procedures) Act 1986 adopting the principles of the 3Rs (Replacement, Reduction and Refinement). The Animal Project Licence was: PPL 2764. The methods proposed under the licence were approved by the Queen’s University Belfast Animal Welfare Ethical Review Body and animals were euthanised using carbon dioxide gas.

### *Fasciola hepatica* material

Italian strain *F*. *hepatica* metacercariae were purchased from Ridgeway Research for generation of transcriptomes. For *in vivo* maintained *F*. *hepatica*, 16 Sprague-Dawley rats were infected with 25 metacercariae each and maintained for 21 days before flukes were recovered from liver tissue. Livers were cubed and incubated at 37°C, 5% CO_2_ in RPMI to allow juvenile fluke to emerge for collection. All juveniles were collected within 2 hours of initial liver processing, washed five times in RPMI to remove liver material and snap frozen for storage at -80°C prior to further processing. Each biological replicate contained 9 juveniles pooled from dissected rats. It was ensured that juveniles for each replicate came from at least 3 different rats collected on 3 separate days to be representative of a general *in vivo* infection. Collected flukes were homogenous in size and there was no obvious correlation between worm counts and fluke size. *In vitro* maintained juvenile replicates were manually excysted as described by McVeigh *et al*. with a prior preparation step of removing the outer casing of metacercariae and bleaching for 2–3 minutes [19]. Excystments of replicates were carried out on consecutive days to generate a total of 1000 juveniles per replicate and to ensure a similar amount of RNA was extracted as from *in vivo* maintained juveniles (determined by juvenile area). Juveniles were maintained in 50% chicken serum and RPMI as described by McCusker *et al*. for 21 days prior to being snap frozen for storage at -80°C prior to further processing [15]. To determine if *in vitro* juveniles can survive anaerobic conditions they were placed in an anaerobic chamber (Don Whitley Scientific, Shipley, UK) set to 37°C and supplied with anaerobic mixed gas (10% CO_2_, 10% H_2_ in N_2_, BOC). To facilitate the maintenance of anaerobic conditions, media was incubated under anaerobic conditions for at least 4 h prior to media changes, which were carried out daily. A time-matched control group was maintained as standard with 5% CO_2_. The pH of anaerobic media was checked to ensure that increased CO_2_ levels did not render it significantly more acidic than media incubated at 5% CO_2_. Trials ran for a duration of 3 weeks, with worm survival being monitored on a weekly basis. Worm death was defined by a total lack of movement and darkened appearance.

### Labelling proliferative nuclei with 5-ethynyl-2-deoxyuridine (EdU)

Visualisation of *Fasciola* proliferative cells from 21 day old juveniles maintained *in vitro* and *in vivo* was achieved by labelling nuclei undergoing DNA-synthesis with 5-ethynyl-2-deoxyuridine (EdU; ThermoFisher Scientific) as described by [15]. Incubations were carried out at the concentration of 500 μM in 50% chicken serum for 24 hours to identify proliferating cells, including possible self-renewal of cells. Juveniles were then flat fixed in 4% paraformaldehyde under coverslips for 10 minutes (*in vitro*) and 40 minutes (*in vivo*) followed by 4-hours free-fixing at room temperature. EdU incubated, fixed juveniles were processed for detection using the Click-iT EdU Alexa Fluor 488 imaging kit, as per kit instructions (ThermoFisher Scientific). Background labelling of all nuclear DNA was achieved using 4′,6-diamidino-2-phenylindole (DAPI). Samples for analysis were mounted in Vectashield (Vector Laboratories) and viewed on Leica TCS SP5 or SP8 confocal microscopes as maximally projected z-stacks generated from 12–15 optical sections from ventral to dorsal surface.

### RNA extraction, library preparation and sequencing

Three biological replicates of *in vitro* maintained and *in vivo* retrieved juveniles were prepared. RNA was extracted using TRIzol reagent (Life Technologies) with an isopropanol precipitation, using glycogen as a carrier. Precipitation was performed overnight at -20°C to maximise small RNA recovery. RNA was DNase treated using the Turbo DNase kit (Ambion) following manufacturer’s instructions and quality control was performed using bioanalyzers to assess for degradation; Qubit for accurate quantification and NanoDrop to indicate sample purity. All accepted samples displayed a 260/280 >2, *i*.*e*. pure RNA with no protein contamination, and a 260/230 >1.8. RNA normalisation, sample library preparation and sequencing were carried out by the Centre for Genomic Research at the University of Liverpool as follows. For RNAseq, RNA was normalised to 1000 ng per sample prior to library preparation. Libraries were prepared using a PolyA selection and the NEBNext Ultra Directional RNA library kit for Illumina to prepare dual indexed, strand specific libraries. Paired end sequencing (2x 150 bp) was performed on the 6 libraries (3 *in vivo*, 3 *in vitro*) using the Illumina HiSeq 4000 platform and generated ~250M mappable reads per sample. For small-RNAseq, libraries were prepared using the NEBNext Small RNA library preparation kit for Illumina and single-end sequencing (1x 50 bp) performed on the HiSeq2500 platform.

### Assembly, annotation and sequence data analyses

Raw sequences were trimmed for the presence of Illumina adapter sequences using Cutadapt (v.1.2.1) [20] and low quality reads using Sickle (v.1.200) [21] with a minimum window quality score of 20. Remaining reads under 10 bp were removed. Final quality control was performed using FastQC (v.0.11.8) [22] with default parameters. The *F*. *hepatica* genome contigs (PRJEB25283) and associated GFF file (PRJEB25283) were downloaded from WormBase ParaSite (WBPS14; https://parasite.wormbase.org/index.html) [23] and annotations were converted to GTF format using Cufflink (v.2.2.2.20150701) [24]. Trimmed FASTQ files were aligned to the *F hepatica* genome (PRJEB25283) using HISAT2 (v.2.1.0) [25] with default parameters and transcripts compiled and counted using StringTie (v.1.3.6) [26,27]. Annotated transcripts were aligned to the genome and combined to generate a gene count file, removing transcript isoforms using StringTie (v.1.3.6). Gene count datasets were filtered to remove non-coding genes and genes with zero read counts. Data were normalised for sequencing depth and RNA composition using DESeq2 (v.1.26.0) [28] package in R (v.3.6.2) [29] with default parameters.

### Annotation and analysis of genes of interest

**Fig 1:**
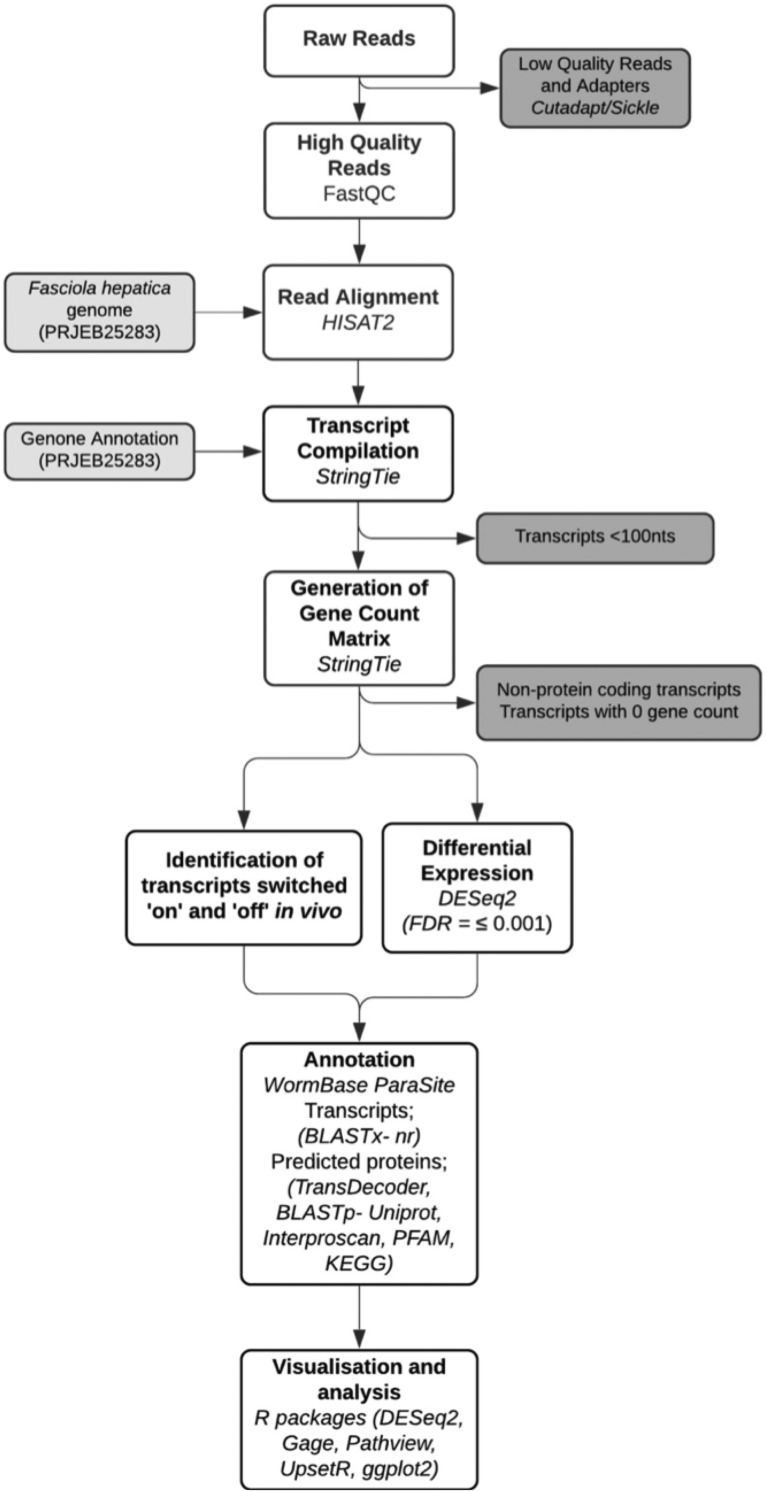
Flow chart of bioinformatics pipeline. Outline of methods used to assemble and analyse transcriptomes. Low quality reads and adapters were removed using Cutadapt (v.1.2.1) and Sickle (v.1.200) and remaining read quality assessed using FastQC (v. 0.11.8) analysis. High quality reads were aligned to the *Fasciola hepatica* transcriptome (PRJEB25283) using HISAT2 (v.2.1.0) and transcripts generated from genome annotation (PRJEB25283) and counted using StringTie (v.1.3.6). Raw gene counts were reviewed for genes of interest (GOI) and analysed for differential expression using R version 3.6.2 and DESeq2 (v.1.26.0) package (FDR <0.001). A core functional annotation pipeline was developed to annotate GOI’s using BLASTx against NCBI non redundant protein (nr) database; predicted proteins generated using Transdecoder; BLASTp predicted proteins against Uniprot reviewed sequence database; functional domain analysis interrogating PFAM and interproscan databases. Graphs were visualised using ggplot2, gate, pathview and upsetR R packages using custom scripts. Chart generated online at: https://app.lucidchart.com. Abbreviations; ivt = *in vitro*, ivv = *in vivo*.

A summary of the annotation pipeline used in this study can be found in Fig 1. Raw gene count datasets generated as previously described were mined for genes absent in one treatment group (across 3 replicates of *in vitro* or *in vivo* transcriptomes) and present in at least 2 replicates of the opposite treatment group with a total read count ≥10. These genes were designated as switched ‘on’ or ‘off’ *in vivo*. Differential expression analysis of protein coding genes was quantified using the DESeq2 (v.1.14.1) [28,30] package in R (v.3.6.2) [29]. A p-value threshold was set for a false discovery rate (FDR) of <0.001 and no threshold was applied for fold change differences. Genes identified using these parameters were described as upregulated or downregulated *in vivo*. Additional datasets were used to provide query sequences for BLAST analysis to support characterisation of neoblast [31], cathepsin [32], neuropeptide [33,34] and G protein coupled receptor [35] target analysis. Metabolic genes were identified by BLAST analysis using reviewed human orthologues obtained from Uniprot (https://www.uniprot.org/). Unannotated differentially expressed genes at this stage were put through the annotation pipeline described in Fig 1. BLASTx analysis of gene sequences was carried out against the NCBI non-redundant protein (nr) database. Transdecoder (v.5.5.0) was used to identify candidate open reading frames and translate genes to proteins for BLASTp analysis against the Uniprot reviewed sequence database. Top hit annotations were collated from both BLAST databases with a p-value ≤0.05. Domain analysis was carried out on predicted proteins using Interproscan (v.5.36–75.0) and PFAM domain analysis using hmmscan functions of HMMER (v.3.1) (p-value ≤0.05). Differentially expressed genes also underwent KEGG pathway analysis to identify important biological pathway differences within datasets. BlastKOALA was used to assign KEGG gene K numbers based on matches with known human pathway genes. R (v.3.6.2) packages DESeq2 (v.1.26.0), gage (v.2.36.0) and pathview (v.1.26.0) were used to analyse results using custom R scripts and determine significant differences (p-value ≤0.05). *F*. *hepatica* GO terms were recovered from WormBase ParaSite (WBPS14; https://parasite.wormbase.org/index.html) [23]. mRNA GO term enrichment was analysed via topGO [36] where FDR <0.05, method = weight01 and statistic = fisher. TopGO enrichment analysis was not performed on miRNA target datasets as this software could not account for individual miRNAs targeting multiple mRNA sequences. Data were visualised using GraphPad Prism (v.8) and R (v.3.6.2) packages, DESeq2 (v.1.26.0), ggplot2 (v.3.3.1) and upsetR. (v.1.4.0) with custom R scripts.

### Identification and differential expression analysis of miRNAs in *in vivo* and *in vitro* maintained *F*. *hepatica*

Small RNA FASTQ files were aligned to a Bowtie index of *F*. *hepatica* genome PRJEB25283 using miRDeep2 (v 2.0.1.2) [37]. miRNAs were defined using the following criteria; present in at least 2 out of 3 replicates for either *in vitro* or *in vivo* transcriptomes, a minimum of 10 reads mapped to the mature sequence, at least 1 read mapped to the star sequence, a significant randfold value and a minimum miRDeep2 score of 5. miRNA naming was consistent with that presented in Herron *et al*. [38]. Differential expression of identified miRNAs was carried out using DESeq2 (version 1.28.1), with an adjusted *p*-value of 0.001 for significance. miRNA target prediction was then carried out using miRanda (version 3.3) [39], with thresholds of minimum pairing score of 150 and maximum free energy score of -20. Predicted targets were refined to those differentially expressed in *in vitro* and *in vivo* mRNA transcriptomes and correlating with miRNA expression, i.e. upregulated in *in vivo* miRNA datasets and downregulated in *in vivo* mRNA datasets, for further interpretation. GO terms associated with predicted miRNA targets were retrieved from WormBase ParaSite (WBPS15) and manually interpreted for frequency (number of times GO term was present in datasets to include data on multiple miRNAs targeting the same mRNA) plotted against log_2_ fold change from previous transcriptome analysis.

## Results

**Fig 2:**
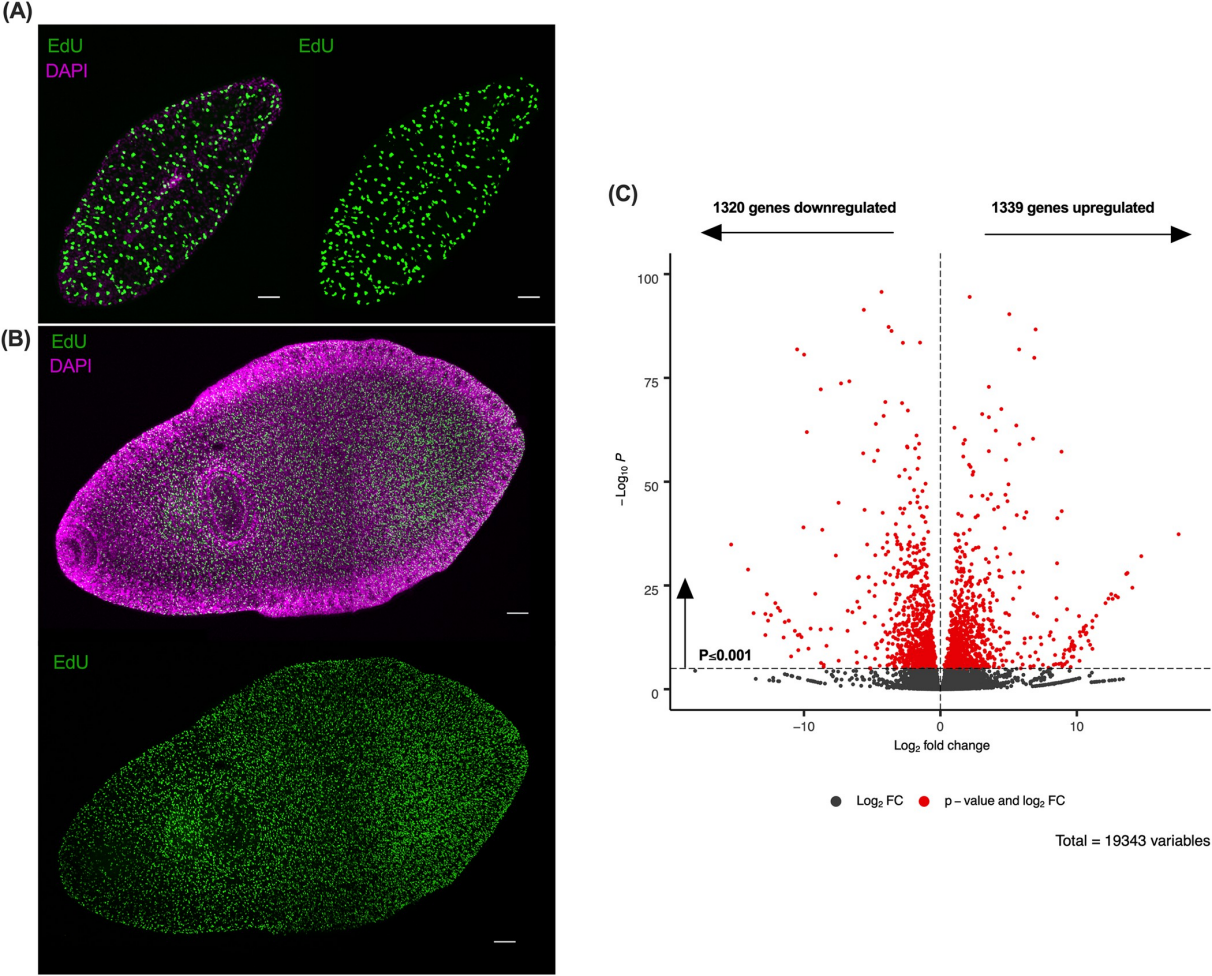
*In vitro* and *in vivo* maintained juvenile liver fluke display differences in growth and cell proliferation. **(A) Cell proliferation represented by EdU+ cells (green) of 21 day old (+1 day labelling) *in vitro* maintained juvenile *Fasciola hepatica*. (B) Cell proliferation represented by EdU+ cells (green) of 21 day old (+1 day labelling) *in vivo* maintained juvenile *F*. *hepatica*.** Confocal Z stack images; green = EdU+; magenta = DAPI; *in vitro* scale bar = 50 μm; *in vivo* scale bar = 100 μm. **(C) Volcano plot of differentially expressed transcripts between *in vitro* and *in vivo* maintained juveniles**. Analysis identified 1339 transcripts upregulated and 1320 transcripts downregulated *in vivo* compared to *in vitro* maintained juveniles with a false discovery rate (FDR) of P≤0.001. Red = significantly differentially expressed genes. Grey = not differentially expressed genes.

Transcriptomes were generated for 21 day old juvenile *F*. *hepatica* maintained *in vitro* and *in vivo* using an Illumina HiSeq RNA sequencing platform. A total of 30,892 transcripts were identified from this analysis which were further refined to define a dataset of 19,343 unique genes for 21 day old juvenile *F*. *hepatica* (S1 and S2 Data; removal of transcript isoforms and genes with non-zero read counts from *in vitro* and *in vivo* assembled transcriptomes) reflective of the greatest depth of sequencing achieved to date for *F*. *hepatica* (average 250 million mapped reads per replicate transcriptome, S1 Table) [40–44]. Functional annotations were assigned to 64% (12,300) of genes based on matches from at least one database interrogated (S2 Table). Differentially expressed genes within these datasets offer insight into the biological differences of juveniles under distinct maintenance conditions and have the potential to identify ways in which *in vitro* culture methods could be improved. Since a primary phenotypic difference between *in vitro* and *in vivo* maintained juveniles is worm size, with age-matched *in vivo* juveniles being ~15-times larger (Fig 2A and 2B), it can be hypothesized that differentially expressed genes also play key roles in juvenile growth and development. These genes have the potential to seed the identification of targets critical to pathogen virulence and establishment within the host. Staining proliferating cells of juveniles in both growth conditions using 5-ethynyl-2-deoxyuridine (EdU) revealed increased EdU+ cells in *in vivo* maintained juveniles (Fig 2B) when compared to *in vitro* counterparts (Fig 2A).

### Differential expression of genes associated with selected cellular processes

DESeq2 was used to analyse differential expression of genes between *in vitro* and *in vivo* treatments. Analysis identified 1339 genes upregulated (6.9%) and 1320 genes downregulated (6.8%) *in vivo* compared to *in vitro* counterparts, amounting to a total of 13.7% of expressed genes being differentially expressed between maintenance treatments (Fig 2C). Functional annotations were assigned to 72% (1923) of differentially expressed genes from at least one hit match from interrogated databases of the annotation pipeline (S3 Table).

**Fig 3:**
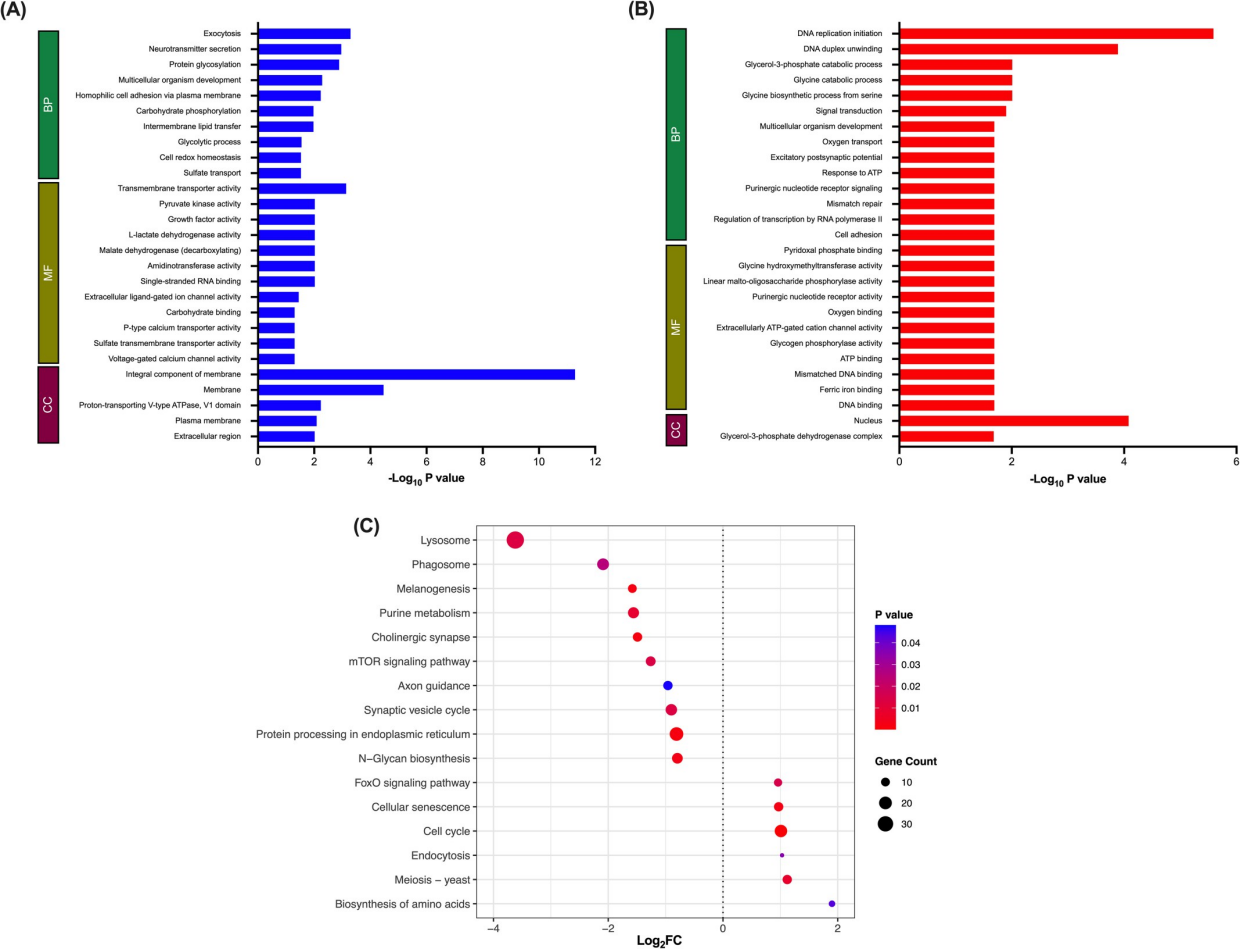
*In vitro* juvenile fluke display enhanced expression of genes required for cell signalling and *in vivo* juvenile fluke display enhanced expression of genes associated with the cell cycle. **(A) TopGO analysis of genes downregulated *in vivo*. (B) TopGo analysis of genes upregulated *in vivo*.** mRNA GO term enrichment was analysed via topGO [36] where FDR <0.05, method = weight01 and statistic = fisher. **(C) KEGG pathway analysis of differentially expressed genes.** KEGG pathways considered significantly differentially expressed as determined by statistical analysis using R (v.3.6.2), gage (v.2.36.0) and pathview (v.1.26.0) packages (P≤0.05). Dotted line = 0 log_2_FC; dots to the right of dotted line represent pathways upregulated *in vivo* and dots to the left of the dotted line represent pathways downregulated *in vivo*. Colour = *p* value; size = gene count.

TopGO analysis of *in vitro* maintained juvenile datasets shows significant upregulation of genes associated with metabolic processes, including glycolysis, L-lactate dehydrogenase activity, malate dehydrogenase activity and pyruvate kinase activity, suggesting some metabolic differences between treatment groups (Fig 3A). A significant increase in the number of genes associated with transmembrane transport, neurotransmitter secretion and ion transport suggests an enhancement of some cell signalling pathways in *in vitro* juveniles when compared to *in vivo* counterparts (Fig 3A). KEGG enrichment analyses corroborates these observations showing a significant downregulation of the synaptic vesicle cycle, axon guidance and, more specifically, cholinergic signalling pathways *in vivo* (Fig 3C). N glycan biosynthesis and protein processing in the endoplasmic reticulum were also significantly downregulated *in vivo* compared to *in vitro* maintained juveniles. Whilst glycosylation has been proposed to play a significant role in host-parasite interaction, our data suggest that glycosylation remains an important protein modification pathway for *in vitro* maintained juveniles in the absence of the definitive host (Fig 3C).

TopGO analysis highlighted an increase in cellular processes associated with DNA replication in datasets of *in vivo* maintained worms with a significant upregulation of genes associated with DNA replication, DNA duplex unwinding and DNA binding compared to *in vitro* maintained worms (Fig 3B). Genes associated with nuclear based cellular components were also upregulated *in vivo* (Fig 3B). Although KEGG pathway analysis showed similar results, it more specifically highlighted a significant upregulation of genes associated with cell cycle, meiosis and cellular senescence pathways *in vivo* (Fig 3C)–all processes associated with cell division.

### Cell cycle- and neoblast-associated genes are upregulated in *in vivo* maintained juveniles

**Fig 4:**
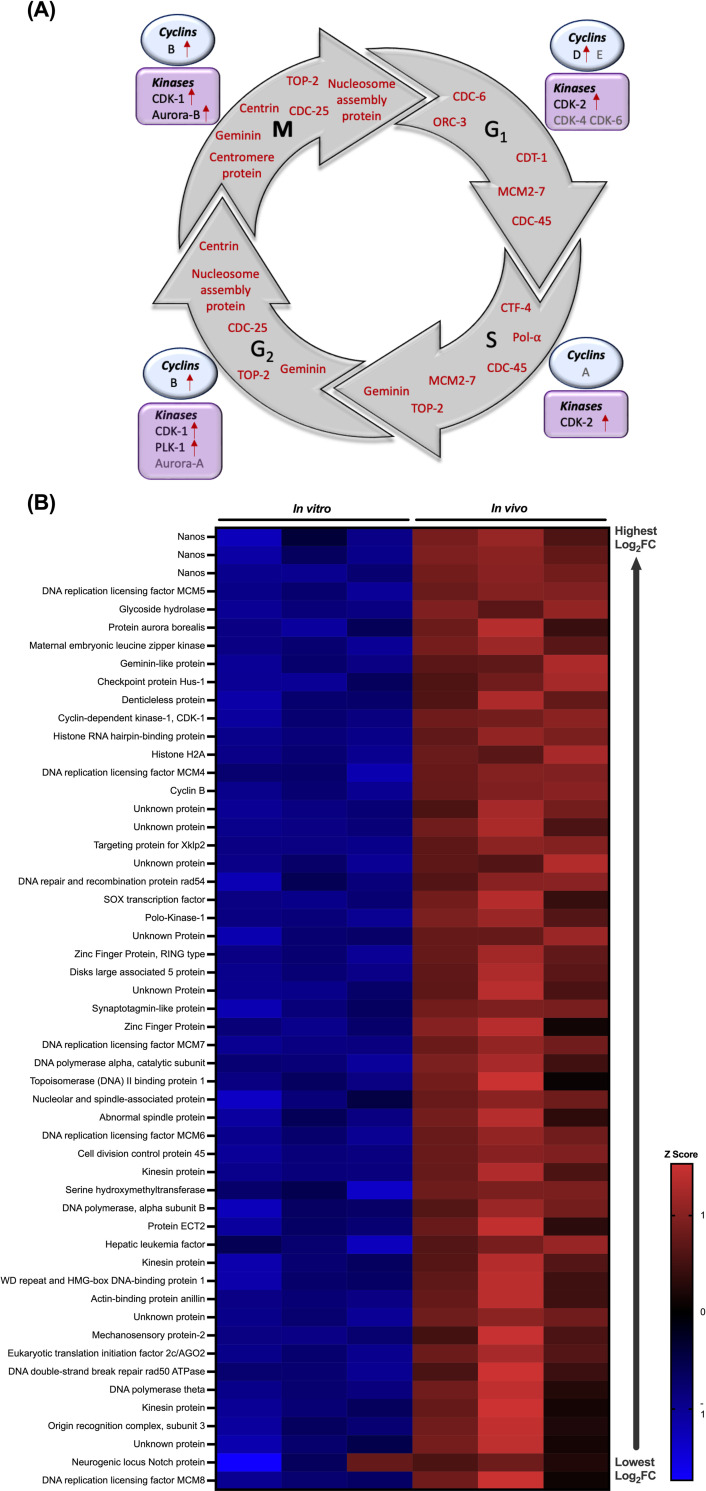
Cell cycle- and neoblast-associated genes are upregulated in *in vivo* maintained juveniles. **(A) Cell cycle associated genes upregulated *in vivo*.** Schematic showing phases and progression of the cell cycle (intermediate (G1), synthesis (S), growth (G2) and mitotic (M) phases). *Fasciola hepatica* homologues of essential cell phase associated genes upregulated *in vivo* are highlighted in red within the cycle. Key cyclins (in blue circles) and kinases (in purple squares) required for each phase of the cell cycle to progress in the human cell cycle are highlighted. *F*. *hepatica* homologues which are upregulated *in vivo* are marked with a red arrow. Abbreviations: CDC(-6, -25, -45), cell division cycle; ORC-3, origin recognition complex subunit 3; CDT-1, chromatin licensing and DNA replication factor 1; MCM2-7, mini-chromosome maintenance complex; TOP-2, DNA topoisomerase 2; Pol-α, DNA polymerase alpha; CTF-4, chromosome transmission fidelity 4; CDK(-1, -2, -4, -6), cyclin-dependent kinase; PLK-1, polo-kinase 1. **(B) Neoblast markers upregulated *in vivo*.** Heatmap showing expression of neoblast markers in *F*. *hepatica* across all replicates of *in vitro* and *in vivo* maintained juveniles. Expression calculated as Z score of transcript per million (TPM) value. Neoblast markers identified as homologues to markers described by Collins *et al*. [31] in *Schistosoma mansoni*.

Eighty nine percent (17,166) of total genes identified were expressed in all replicates of *in vitro* and *in vivo* juveniles (Sheet A in S4 Table; 3x *in vitro = in vitro* 1–3; 3x *in vivo = in vivo* 1–3). In addition to differentially expressed genes, those genes which are present in only one treatment group, *i*.*e*. genes considered switched ‘on’ or ‘off’ *in vivo* are also genes of interest for understanding mechanisms of juvenile growth and development. A significant proportion of the genes only identified *in vivo* are associated with cell cycle processes and transcription regulation including cyclin-dependent kinase, cyclin, centromere protein, nucleosome assembly protein, transcription factors and zinc finger proteins (Sheet B in S4 Table). Increased expression of these genes in *in vivo* datasets is consistent with increased cell proliferation and turnover in these juveniles. Closer examination of all cell cycle-associated genes shows a high proportion of these genes upregulated *in vivo* across all cell-cycle stages, corroborating increased cell division in these juveniles (Fig 4A). In particular, all components of the highly conserved MCM2-7 complex and cell division cycle genes (*cdc-6*, *cdc-45*, *cdc-25*) known to tightly regulate key components of DNA replication (Fig 4A). Key regulators of cell cycle progression, including cyclins and cyclin-dependent kinases (CDKs), are also significantly upregulated suggesting cell cycle activities are higher *in vivo* (Fig 4A). The kinases, polo-like kinase-1 (*plk-1*) and aurora-b kinase (*aurkb*), thought to ensure accurate chromosomal segregation, were also upregulated *in vivo*, which would appear to be consistent with the phenotypic observations (Fig 4A). To characterise further the relationship between growth/development and neoblast-like stem cells, differential expression analysis considered the 128 neoblast-like cell markers identified in *S*. *mansoni* [31]. Of these genes, 108 were found to have orthologues in the *F*. *hepatica* genome and 53 were shown to be differentially expressed and upregulated in *in vivo* maintained juveniles (S5 Table), consistent with the altered stem cell dynamics needed to drive faster growth and development in these juveniles (Fig 4B and S5 Table). Amongst the known neoblast markers significantly upregulated in the *in vivo* juveniles were *nanos* (3 genes), a key post-transcriptional regulator and the transcription factor *sox-1*, both previously identified as being essential for neoblast proliferation in flatworms [31,45,46] (Fig 4B). These data offer new insight into important genes regulating essential mechanisms of *F*. *hepatica* growth and development and provide a starting point for functional validation as potential control targets.

### Protease profile dynamics differ within the *in vitro* and *in vivo* maintained juveniles

**Fig 5:**
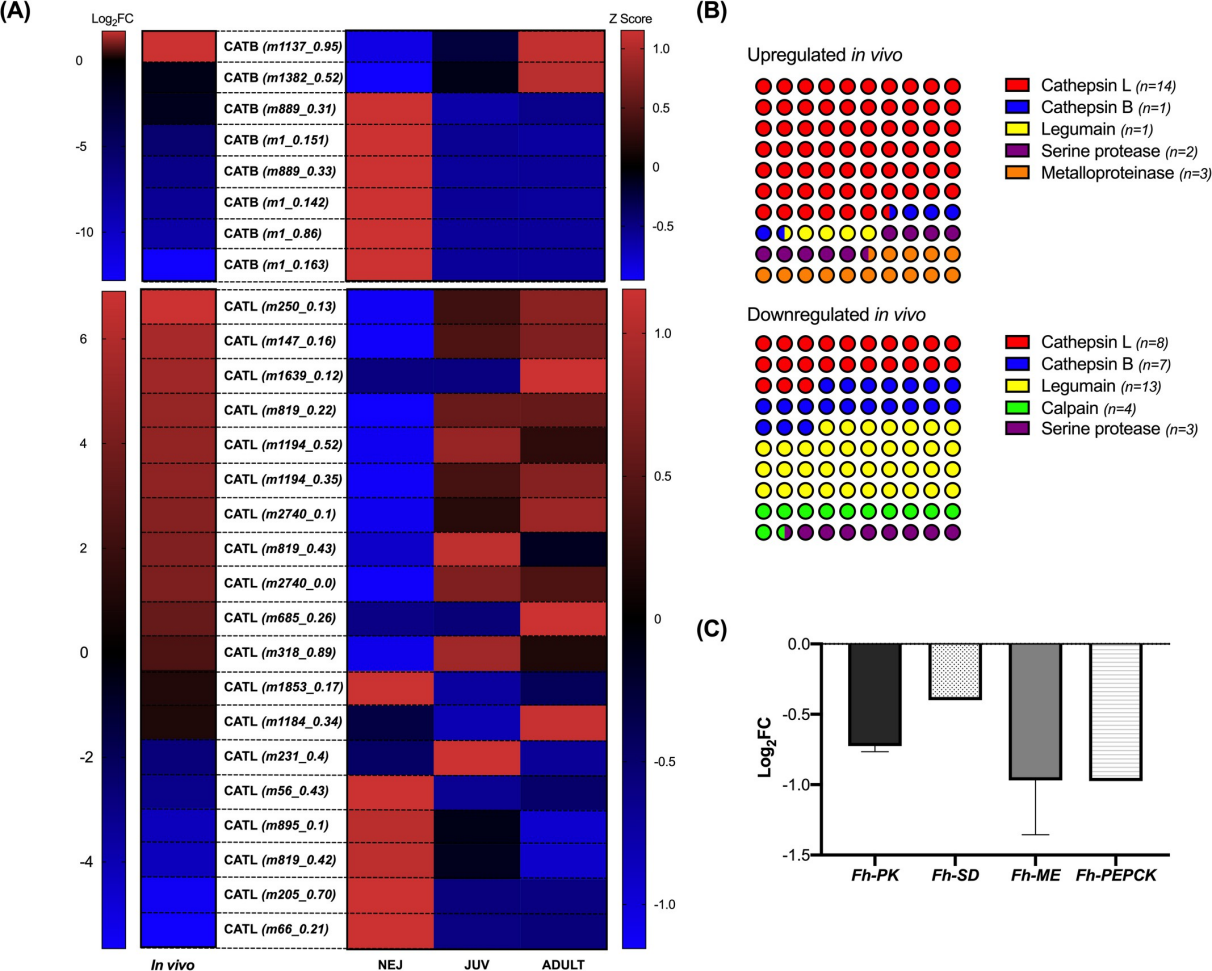
*In vivo* and *in vitro* juvenile liver fluke show divergent expression of proteases and metabolic enzymes. **(A) Cathepsin expression profile across newly excysted juvenile (NEJ), 21 day old juvenile (JUV) and adult *Fasciola hepatica* life stages compared to expression in *in vitro* and *in vivo* maintained *F*. *hepatica* juveniles.** Differential expression of cathepsin B (CATB) and cathepsin L (CATL) encoding transcripts in *in vivo* and *in vitro* maintained *F*. *hepatica* juveniles (log_2_FC) and across *F*. *hepatica* life stage transcriptomes generated by Cwiklinski *et al*. [41] (NEJ, JUV, ADULT; Z score of fragments per kilobase of transcript per million mapped reads, FPKM). Abbreviated gene IDs in brackets refer to gene IDs in S6 Table. Note that only the expression of genes annotated in both Cwiklinski *et al*. [41] and our datasets were analysed, novel genes could not be analysed due to their absence from the original genome annotation. **(B) Protease/peptidase profiles of *in vitro* and *in vivo* maintained *F*. *hepatica* juveniles.** Proportion diagram showing expression of proteases in *in vitro* and *in vivo* maintained juveniles. Individual gene counts (n =) are shown beside each protease group. **(C) Downregulation of key enzymes of aerobic and anaerobic carbohydrate metabolism in *in vivo* maintained *F*. *hepatica* juveniles.** Expression (log_2_FC) of key enzymes associated with metabolism in *in vivo* maintained *F*. *hepatica* juveniles. *Fh*-PK = pyruvate kinase and Fh-SD = succinate dehydrogenase are key enzymes of aerobic metabolism via Kreb’s cycle; *Fh*-ME-malic enzyme is a key enzyme of aerobic acetate production; *Fh*-PEPCK = phosphoenolpyruvatecarboxykinase, key enzyme of malate dismutation pathway.

Cathepsin (CAT) proteases represent >80% of the proteins secreted by adult liver fluke [32] and are of particular interest due to their role at the host parasite interface and potential in vaccine development [32,47–49]. CATs display marked temporal changes in expression during juvenile fluke development associated with altering proteolytic requirements of the parasite as it migrates within the mammalian host [32,45]. 34 potential CATs (23 CATL and 11 CATB) have been previously described within the *F*. *hepatica* genome [32]. Our datasets, suggest that *F*. *hepatica* in fact express 38 individual CAT (24 CATL and 14 CATB) proteins (S6 Table). Of the 38 CATs identified from this analysis, 31 genes were differentially expressed suggesting that CAT profiles of *in vitro* and *in vivo* maintained juveniles are considerably different (Fig 5A). 21 CATLs were differentially expressed, of which 13 were upregulated *in vivo* (Fig 5A (left) and S6 Table). Maker-scaffold10x_250_pilon-snap-gene-0.13 was identified as the most upregulated CAT *in vivo* (log_2_FC = 17.46; padj = 1.22E-113) and was found to be a CATL1 protein. There was a general trend of CATL1 genes being upregulated *in vivo*. SignalP (v.5.0) analysis of upregulated cathepsins showed 80% contained a signal peptide, suggesting they may be secreted at the host-parasite interface (S6 Table). CATL encoding genes downregulated *in vivo* are thought to belong to clade 5, suggesting these cathepsins in particular are more important in biology associated with *in vitro* maintenance (S6 Table). Little is known of the function of clade 5 CATLs, however, these data suggest a function at earlier stages of the liver fluke life cycle. Ten CATB proteins were differentially expressed, 9 of these were downregulated *in vivo* (Fig 5A (left) and S6 Table); 2 novel CATB proteins not previously annotated were identified (MSTRG.21893 and MSTRG.6836). The downregulation of CATB genes *in vivo* was also correlated with a downregulation of legumain regulatory peptidases, likely relating to the *trans*-activation of CATB proteases by legumains (Fig 5B). These data suggest CATB proteases are more important to the biology of *in vitro* maintained juveniles, whilst CATL proteases are more significant in the biology of *in vivo* maintained juveniles, likely due to host interactions. Differences in cathepsin expression may relate to developmental differences between the two treatment groups–indeed, a switch from CATB protease expression to CATL expression has been linked with the development of *F*. *hepatica* juveniles *in vivo* [32,45]. To investigate this hypothesis, differential expression of CAT genes annotated in the genome PRJEB25283 (i.e. not including novel cathepsins identified in this study; 21 CATL and 8 CATB genes) was correlated with expression in previously published life stage transcriptomes, with a specific focus on newly excysted juvenile (NEJ), 24 hour juvenile and adult datasets (Fig 5A). These data show a general trend of CATs upregulated *in vivo* showing greater expression in the juvenile and adult stages of development, whilst the CAT genes downregulated *in vivo* showed greater expression in the 24 hour NEJ stage of development (Fig 5A). These observations are consistent with a developmental delay in the *in vitro* maintained juveniles compared to their age matched *in vivo* juveniles, suggesting *in vitro* maintained juveniles are closer in development to 24 hour NEJs or are unable to switch CAT expression to those expressed later in development in the absence of host-specific cues. Other protease expression dynamics observed included an increased proportion of metalloproteinases in the *in vivo* juveniles, likely to aid in host tissue degradation (Fig 5B). There was an increased proportion of calpain proteases in *in vitro* maintained juveniles, consistent with observations in *Schistosoma* spp. where they are important for early juvenile migration and host evasion due to their role in immune defence [50].

### Metabolic differences between *in vitro* and *in vivo* maintained *F*. *hepatica* juveniles

Parasitic flatworms rely heavily on carbohydrate substrates for energy metabolism [51]. *F*. *hepatica* have been shown to switch from aerobic energy metabolism using the Tricarboxylic Acid Cycle (TCA) pathway, via aerobic acetate production, to anaerobic dismutation [41,51] as they transition from free living to parasitic life stages. *In vitro* cultured juveniles are maintained under aerobic conditions long term supplemented with 5% CO_2_, whilst 21 day *F*. *hepatica* juveniles burrowing through the liver parenchyma are thought to be undergoing a switch to predominantly anaerobic metabolism. BLAST analysis was used to broadly identify key components of aerobic and anaerobic metabolism within the liver fluke genome (S7 Table). Correlating with topGO analysis, some key enzymes (pyruvate kinase, Fh-PK; succinate dehydrogenase, Fh-SD; malic enzyme, Fh-ME; phosphoenolpyruvate carboxykinase, Fh-PEPCK) associated with all pathways of carbohydrate metabolism (aerobic and anaerobic) were downregulated *in vivo*, suggesting enhanced metabolic activities, both aerobic and anaerobic, in *in vitro* maintained juveniles (Fig 5C). However, on closer examination, few other enzymes associated with metabolism were considered significantly differentially expressed, suggesting little difference in the metabolic processes of *in vitro* and *in vivo* maintained juveniles (S7 Table). The upregulation of lactate dehydrogenases in *in vitro* datasets may suggest these juveniles are generating greater levels of lactate metabolic waste than *in vivo* juveniles (S7 Table). To define this further, *in vitro* maintained juveniles were maintained in an anaerobic chamber, removing the ability to undergo aerobic respiration. These juveniles were unable to grow and showed declining health across a two week period when compared to juveniles maintained under aerobic 5% CO_2_ conditions (S1 Fig). After 3 weeks, significant death (87.4%) resulted in termination of the experiment.

### N-glycan biosynthesis and processing enzymes are downregulated *in vivo*

KEGG pathway analysis highlighted the downregulation of genes associated with N-glycan biosynthesis and processing *in vivo* (Fig 3D). On closer examination of protein glycosylating genes described by McVeigh *et al*. [52], downregulation *in vivo* is particularly associated with components of the oligosaccharyl transferase (OST) complex involved in the *en-bloc* transfer of glycans to proteins [53], including catalytic core domain proteins STT3A/STT3B and RPN-1/-2 proteins (S2 Fig). Glycans are commonly linked to roles in host parasite interactions [54,55] and the high expression of glycosylating enzymes associated with glycan biosynthesis in *in vitro* maintained worms suggests that aspects of host-parasite interactions are sustained *in vitro* even in the absence of a host. It is important to note that there was no differential expression of O-linked glycosylation genes, again thought to correlate with host-parasite interactions [54,55].

### Components of the *F*. *hepatica* nervous system are downregulated *in vivo*

**Fig 6:**
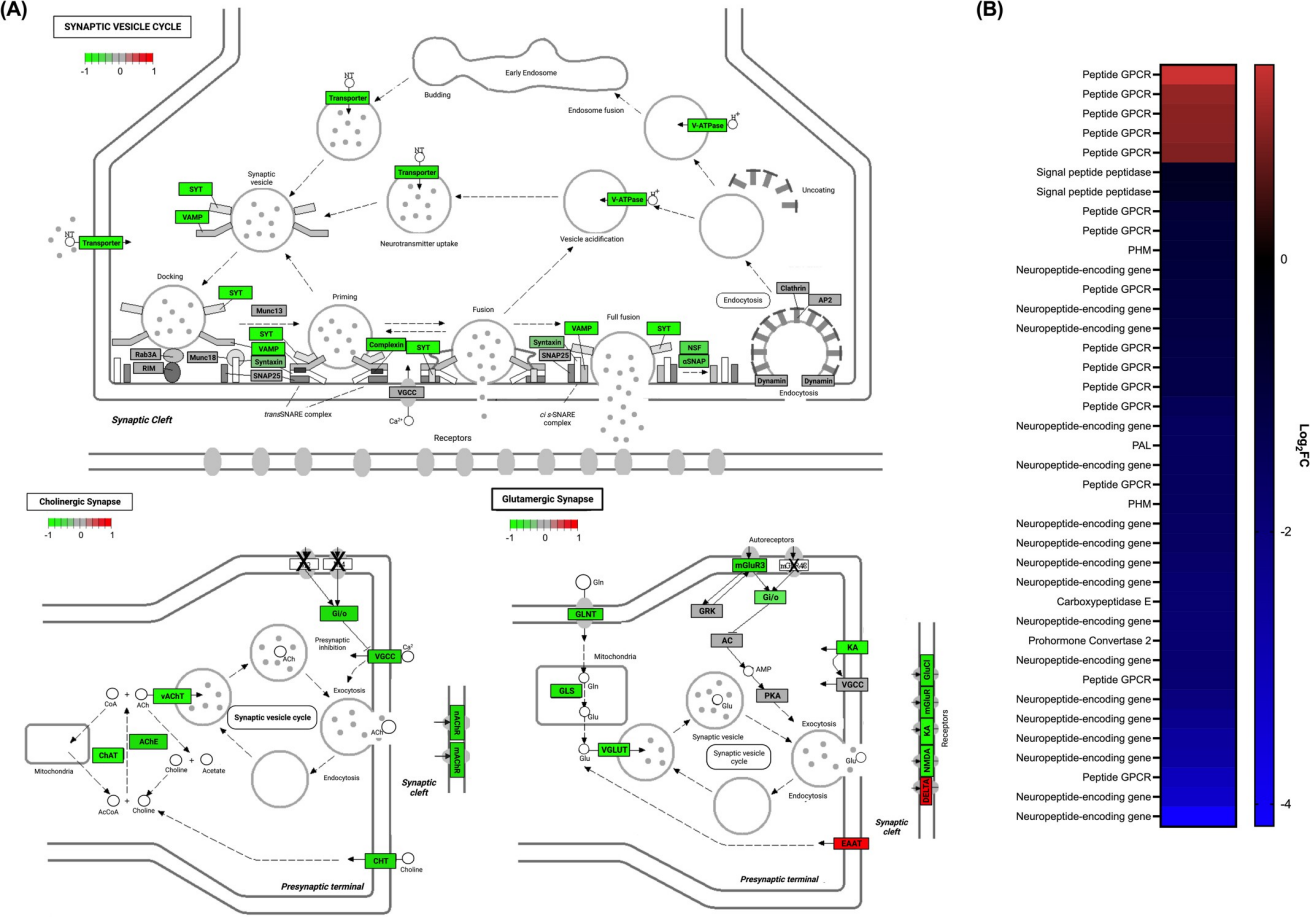
Key neuronal signalling pathways are downregulated in fast growing *in vivo* juvenile liver fluke. **Components of classical neurotransmission are downregulated in *in vivo* maintained juvenile liver fluke (A).** Differential expression of nervous system components of the synaptic vesicle cycle, cholinergic signalling and glutamatergic signalling. *Fasciola hepatica* homologues of human components enclosed in rectangular boxes. Green = downregulated *in vivo*, red = upregulated *in vivo*, grey = not differentially expressed, X = not present in *F*. *hepatica* genome (PRJEB25283). Abbreviations: SYT, synaptotagmin; VAMP, vesicle associated membrane protein; Rab3a, ras-related protein; RIM, regulating synaptic membrane exocytosis protein; MUNC(-13, -18), syntaxin binding protein; SNAP25, synaptosome associated protein; VGCC, voltage-gated calcium channel; NSF, N-ethylmaleimide sensitive factor; αSNAP, NSF associated protein; AP2, adaptor related protein complex 2; ChAT, choline O-acetyltransferase; AChE, acetylcholinesterase; vAChT, vesicular acetylcholine transporter; CHT, high affinity choline transporter; Gi/o, guanine nucleotide-binding protein; mAChR, muscarinic acetylcholine receptor; nAChR, nicotinic acetylcholine receptor; GLNT, amino acid transporter; GLS, glutaminase; vGLUT, vesicular glutamate transporter; GRK, G protein-coupled receptor kinase; AC, adenylate cyclase 1; PKA, protein kinase A; EAAT, glial high affinity glutamate transporter; mGluR, muscarinic glutamate receptor; GluCl, glutamate-gated chloride channel; KA, kainate type ionotropic glutamate receptor; NMDA, NMDA type ionotropic glutamate receptor; DELTA, delta type ionotropic glutamate receptor. **Components of neuropeptidergic signalling pathway are downregulated in *in vivo* juvenile liver fluke (B).** Heatmap showing differentially expressed genes associated with neuropeptidergic signalling in *Fasciola hepatica*.

TopGO and KEGG pathway analysis highlighted a significant downregulation of nervous system components *in vivo*. Flatworms employ both classical and neuropeptidergic neurotransmitters for neurotransmission [56–58] and both displayed markedly reduced expression in the *in vivo* juveniles (Fig 6).

The synaptic vesicle cycle is responsible for packaging and releasing neurotransmitters from the synapse to modulate neurotransmission [59]. All major components of this pathway were significantly downregulated *in vivo* suggesting an increased rate of neurotransmitter release, and as a result, classical neurotransmission communication in *in vitro* maintained juveniles (Fig 6A; top). Further examination revealed downregulation of the cholinergic signalling pathway and a noteworthy partial downregulation of the glutamate-signalling pathway, emphasising the importance of these two pathways to the biology of *in vitro* maintained juveniles (Fig 6A; bottom). Although classical neurotransmission is most commonly associated with modulating neuromuscular control in parasitic flatworms [60], these data suggest a novel role in growth and development. No pathway genes associated with dopamine or tyramine/octopamine signalling were found to be differentially expressed, whilst only tryptophan hydroxylase and serotonin reuptake transporter from the serotonin signalling pathway were found to be significantly downregulated in *in vivo* maintained juveniles. It should also be noted that none of the putative serotonin G protein coupled receptors identified by McVeigh *et al*. [35] were differentially expressed. Serotonin has been highlighted as an essential component of the *F*. *hepatica* nervous system and, alongside dopamine, is thought to have a core role in neuromuscular control [61–63]. Stable expression of a small cohort of neurotransmitter signalling components, including from the serotonin signalling pathway, between *in vitro* and *in vivo* maintained juveniles suggests some core functioning of the liver fluke nervous system was not changed by maintenance treatment.

Interestingly, major components of neuropeptide signalling were also downregulated *in vivo* with the entire neuropeptide processing pathway displaying reduced expression [57]. Genes encoding two signal peptidases, prohormone convertase-2 (*PC-2*), carboxypeptidase E (*CPE*), peptidylglycine α-hydroxylating monooxygenase (*PHM*) and peptidyl-alpha-hydroxyglycine alpha-amidating lyase (*PAL*) were all downregulated *in vivo* (Fig 6B). The downregulation of neuropeptide processing suggests neuropeptides are being produced at a decreased rate *in vivo* compared with *in vitro* maintained juveniles, suggesting neuropeptide signalling displays changing expression dynamics during development. We identified 34 neuropeptide (*npp*)-encoding genes in the *F*. *hepatica* genome (PRJEB25283, WBPS14) and 17 were differentially expressed between maintenance treatments; all 17 were significantly downregulated in *in vivo* juveniles compared to *in vitro* juveniles (S8 Table), corroborating the hypothesis that neuropeptide signalling is increased in *in vitro* maintained juveniles and may play a previously undescribed role in modulating liver fluke growth and development. 6 *npp*-encoding genes were downregulated *in vivo* with a log_2_FC <-2 and are of particular interest as potential modulators of growth and development in *F*. *hepatica* juveniles. Two of the most significantly downregulated *npp*-encoding genes are NPF/NPY neuropeptides, homologous to vertebrate NPY neuropeptides (S8 Table) [33,58]. McVeigh *et al*. [35] identified 35 *F*. *hepatica* G-protein coupled receptors (GPCRs). Combining available genomic and transcriptomic datasets suggests *F*. *hepatica* expresses 44 peptide GPCRs, of which 15 are differentially expressed (S8 Table). Ten peptide GPCRs were downregulated *in vivo* and one of these was previously hypothesized to be an NPY receptor. Further, 5 peptide GPCRs were downregulated in the *in vitro* juveniles, corroborating KEGG pathway analysis which suggests GPCR signalling is relatively higher in the *in vitro* maintained juveniles. These observations support the hypothesis that selected classical neurotransmitters and neuropeptides act to modulate growth and development of *F*. *hepatica* juveniles, warranting further experimental exploration.

### MicroRNAs (miRNA) are differentially expressed in *in vitro* and *in vivo* maintained juveniles

**Fig 7:**
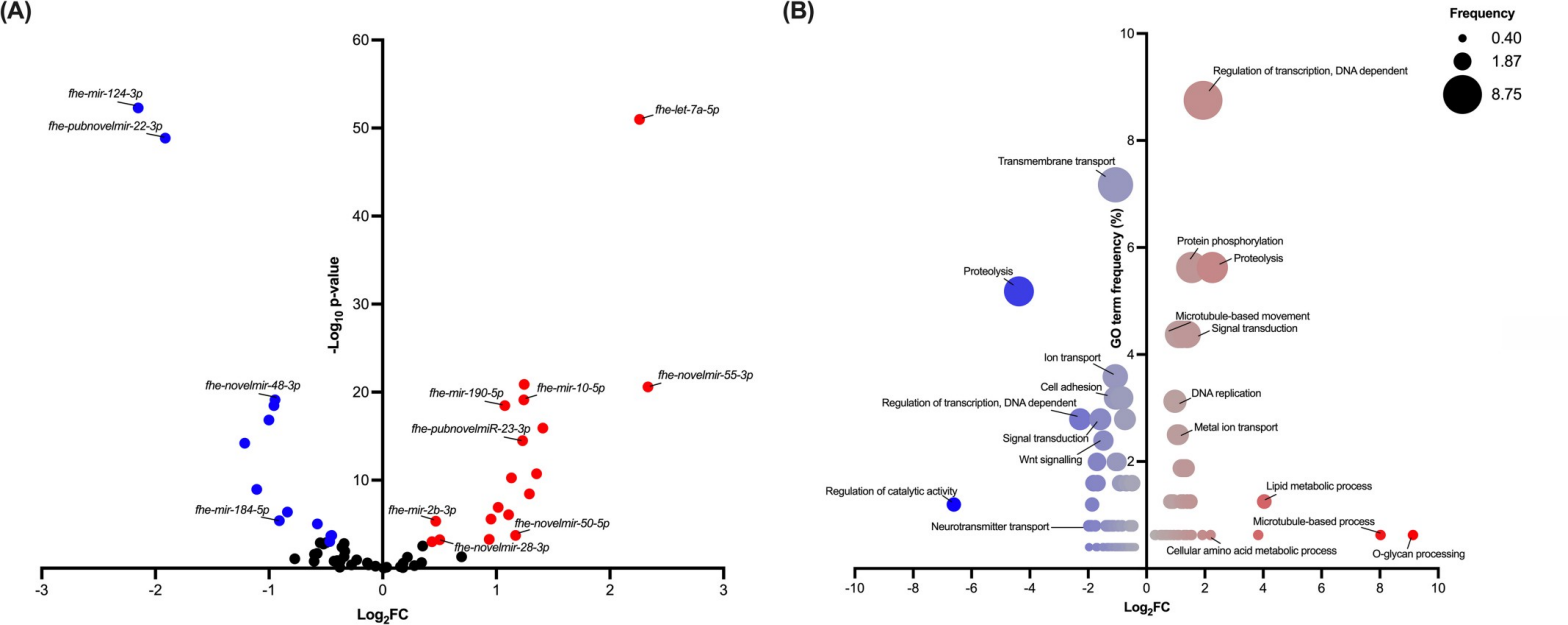
Differences in miRNA profiles of *in vitro* and *in vivo* juveniles support their role in liver fluke developmental processes. **(A) Differential expression of micro (mi)RNA sequences in *in vitro* and *in vivo* maintained 21-day old *Fasciola hepatica* juveniles.** Analysis identified 31 differentially expressed miRNAs between treatment groups (false discovery rate; P≤0.001). Red points highlight significantly upregulated miRNAs *in vivo*, whereas blue points highlight significantly downregulated miRNAs *in vivo*. The two most significantly upregulated miRNAs (*fhe-let-7a-5p*, first identified by Fromm *et al*. [65] and *fhe-novelmir-55-3p*), downregulated miRNAs (*fhe-mir-124-3p*, first identified by Fontenla *et al*. [66] and *fhe-pubnovelmir-22-3p* first identified by Fromm *et al*. [65]) and miRNAs identified as associated with Wnt signalling (*fhe-mir-10-5p*, *fhe-mir-184-5p*, *fhe-mir-190-5p*, *fhe-mir-2b-3p*, *fhe-pubnovelmir-22-3p*, *fhe-pubnovelmiR-23-3p*, *fhe-novelmir-48-3p*, *fhe-novelmir-50-5p* & *fhe-novelmir-28-3p*) are labelled. **(B) Gene ontology (GO) terms associated with biological processes of differentially expressed miRNA predicted gene targets.** GO terms retrieved from WormBase ParaSite (WBPS15) for predicted miRNA targets upregulated or downregulated *in vivo*. Number of individual GO terms associated with biological processes (percentage frequency of total GO terms) plotted alongside average log_2_ fold change of predicted transcripts from differential expression analysis of *in vitro* and *in vivo* maintained *F*. *hepatica* juveniles. Bubble colour intensity = transcript expression (log_2_FC; red = upregulated mRNAs; blue = downregulated mRNAs); bubble size = GO term frequency (percentage gene counts).

miRNAs function in post-transcriptional regulation of gene expression [64–67]. To determine the role of miRNAs in the regulation of transcripts associated with growth and development, miRNA sequencing was carried out on RNA extracted from *in vitro* and *in vivo* maintained juvenile liver fluke. This study identified 103 miRNAs in liver fluke, including 14 novel miRNAs (Sheet A in S9 Table) and 89 miRNAs reported in previous studies on *F*. *hepatica* [38, 64–67]. Recently, Herron *et al*. [38] highlighted considerable redundancy across published miRNAs and moved to refine the current cohort of *F*. *hepatica* miRNAs by removing duplicate sequences of high similarity and retaining the longer sequence as individual miRNAs. Applying this approach to our dataset refines our final miRNA dataset to 89 miRNAs (75 previously published [64–67]; 14 novel). Of the 89 total miRNAs described for *F*. *hepatica*, 31 were differentially expressed between *in vivo* and *in vitro* maintained juveniles; 18 miRNAs were significantly upregulated in *in vivo* juveniles and 13 were significantly downregulated in *in vivo* juveniles, suggesting a role for these miRNAs in transcriptional regulation across maintenance conditions (Fig 7A and Sheet A in S9 Table). The two most significantly upregulated miRNAs were *fhe-let-7a-5p*, first identified by Fromm *et al*. [65] and *fhe-novelmir-55-3p*. The two most significantly downregulated miRNAs were *fhe-mir-124-3p*, first identified by Fontenla *et al*. [66] and *fhe-pubnovelmir-22-3p* first identified by Fromm *et al*. [65]). Gene ontology (GO) term analysis of predicted gene targets for differentially expressed miRNAs shows the increased expression of mRNA targets associated with transcriptional regulation, microtubule-based processes and DNA replication, whereas predicted mRNA targets associated with transmembrane transport, ion transport and signal transduction were downregulated in *in vivo* juveniles (Fig 7B). These data correspond with mRNA analysis and suggest these processes are at least partially regulated by miRNAs in liver fluke. Notably, Wnt signalling was identified as a process likely to be impacted by differentially expressed miRNAs with upregulated miRNAs (*fhe-mir-10-5p*, *fhe-mir-190-5p*, *fhe-mir-2b-3p*, *fhe-pubnovelmiR-23-3p*, *fhe-novelmir-50-5p*, *fhe-novelmir-28-3p*) predicted to target Wnt-associated genes that displayed reduced expression in *in vivo* maintained juveniles (Fig 7 and Sheet B in S9 Table). Five out of eight Wnt-associated genes downregulated in *in vivo* juveniles were predicted as regulated by miRNAs (2 Wnt proteins, maker-scaffold10x_735_pilon-snap-gene-0.38 & maker-scaffold10x_254_pilon-augustus-gene-0.12; 1 secreted frizzled-related protein, maker-scaffold10x_405_pilon-snap-gene-0.20; 2 frizzled GPCRs, maker-scaffold10x_541_pilon-snap-gene-0.38 & maker-scaffold10x_944_pilon-snap-gene-0.48). Three downregulated miRNAs (*fhe-novelmir-48-3p*, *fhe-mir-184-5p* & *fhe-pubnovelmir-22-3p*) were also predicted to target low-density lipoprotein receptor-related protein (LRP) 5/6 (maker-scaffold10x_442_pilon-snap-gene-0.18), a Wnt-associated gene upregulated in *in vivo* maintained juveniles (Fig 7 and Sheet B in S9 Table).

Two downregulated miRNAs were more significantly differentially expressed than others, *fhe-mir-124-3p* identified by Fontenla *et al*. [66] and *fhe-pubnovelmir-22-3p* identified by Fromm *et al*. [65] (Fig 7A). In contrast *fhe-let-7a-5p*, first identified by Fromm *et al*. [65] and considered a highly conserved miRNA, was the most significantly upregulated miRNA in *in vivo* maintained juveniles, whilst *fhe-novelmir-55-3p* identified from this study was the most upregulated miRNA (log_2_FC = 2.33) in the *in vivo* maintained juveniles (Fig 7A). Analysis suggested that these four miRNAs play a significant role in regulating the expression of genes associated with *in vitro* and *in vitro* maintenance. Differentially expressed predicted targets for these miRNAs include transcription factors, cell cycle proteins, apoptosis inhibitors, growth factors, metabolic and glycosylating enzymes (Sheet B in S9 Table).

## Discussion

To date, this is the most in depth transcriptomic study of *F*. *hepatica* juveniles [40–44], providing crucial datasets for understanding the biology of this pathogenic stage, improving understanding and informing drug target identification and validation efforts.

13.7% of genes were considered differentially expressed in this study, with >86% of genes expressed at similar levels in *in vitro* and *in vivo* maintained juveniles. This suggests that core biological functioning is consistent in juveniles under the two growth conditions and despite the size differences observed between juveniles, the current *in vitro* maintenance platform supports relevant biological processes [15]. This corroborates previous observations that *in vitro* maintained juveniles develop phenotypic attributes consistent with *in vivo* developing juveniles [15], supporting the value of the *in vitro* functional genomics platform for initial drug target validation studies prior to the use of animal models of infection. Although *in vitro* and *in vivo* juvenile biology appear largely consistent, it is clear that parasites are highly adaptive and respond rapidly to changing external conditions [68]. It is likely that *in vitro* maintained juveniles are not exposed to host-related triggers that enhance growth/development dynamics as observed in the *in vivo* juveniles and a better understanding of these cues may be key to improving current *in vitro* culture methods.

Transcriptome differences between the *in vitro* and *in vivo* juveniles were consistent with the observed differences in the level of cell proliferation. Genes associated with cell proliferation and known neoblast markers were significantly upregulated *in vivo*, correlating with larger juvenile size. Neoblasts have previously been associated with growth and development of many flatworm species, including *F*. *hepatica* and *Schistosoma mansoni* [15,31,69–73]. In the cestode *Echinococcus multilocularis*, stem cells were shown to be the only cells driving metacestode growth and regeneration [72]. Again, this suggests greater cell proliferation *in vivo* leading to more rapid development of these juveniles. In addition to the role in growth and development, neoblast proliferation has also been associated with rapid tegumental renewal and tissue repair of parasites *in vivo*, suggesting the importance of these cells to host-parasite interactions [74]. The tegument is the barrier between host and parasite such that its rapid renewal allows parasites to counter host immune responses through active evasion and/or repair of immune response-associated damage [74]. Clearly, the upregulation of neoblast-associated genes *in vivo* may partly relate to the demands of immune challenge and *in vivo* survival, challenges not faced during *in vitro* culture. It is noteworthy that a higher rate of neoblast proliferation was associated with the mitigation of tissue damage and crucial to *S*. *mansoni* survival *in vivo* [75].

The slower development of *in vitro* juveniles is also reflected in the expression patterns of CATL and CATB proteases. It is well documented that juvenile flukes undergo a developmental shift from CATB expression to CATL expression as they develop from NEJs in the duodenum to adult liver fluke in the bile duct [32,47,48]. This is thought to happen early in the developmental life cycle [76] reflecting the changing protease requirements associated with feeding and tissue degradation [32]. Our transcriptomic datasets show greater expression of CATB proteases in the *in vitro* juveniles, reflective of early stage *F*. *hepatica* juveniles, whilst CATLs are more prominent in the *in vivo* juveniles, characteristic of later stages of development. It is likely that a developmental delay of *in vitro F*. *hepatica* is responsible for the differences observed between *in vitro* and *in vivo* maintained juveniles, however, it is also possible that host-derived triggers during migration drive the changing cathepsin expression profiles seen in the *in vivo* juveniles. These data encourage further studies comparing earlier *in vivo* juveniles and later *in vitro* juveniles to see if matching cathepsin expression profiles could be a proxy for the alignment of *in vivo* and *in vitro* developmental stage. In addition to cathepsins, other proteases thought to play roles in host-parasite interactions, such as calpains, were also evident in the *in vitro* datasets [50]. Somewhat surprisingly, the proposed immunomodulatory protein helminth defence molecule (HDM-1), was not differentially expressed between the *in vitro* and *in vivo* maintained juveniles [77–79]. Indeed, the gene encoding HDM-1 had a raw read count of >1 million in both treatment groups.

Glycan biology is also considered key for host-parasite interactions [54,55]. Our analysis revealed the consistent expression of proteins associated with O-linked glycosylation and the higher expression of N-linked processing enzymes in *in vitro* maintained juveniles. The specific relationship between N-glycans and *in vitro* maintained juveniles is unknown, but it is perhaps due to the availability of glucose in chicken serum, an important precursor of the N-linked glycan biosynthetic pathway [80,81]. It is also possible that these specific glycans are more important for some aspects of biology associated with *in vitro* culture or are associated with host-parasite interactions at earlier stages of parasite development. These observations suggest that *in vitro* cultured *F*. *hepatica* juveniles have utility in informing aspects of the biology of genes involved in host-parasite interplay. No significant metabolic differences were observed between *in vitro* and *in vivo* juveniles, although some key genes associated with carbohydrate metabolism (aerobic and anaerobic) were upregulated in *in vitro* maintained juveniles. It is possible that this observation relates to the fact that chicken serum has a glucose content twice the level of that seen in mammals [81]; the increased expression of genes associated with carbohydrate metabolism may reflect the juveniles taking advantage of the higher substrate availability. During their development from NEJs to adult fluke, juvenile *F*. *hepatica* are reported to display a shift from aerobic to anaerobic carbohydrate metabolism. Early studies by Tielens *et al*. showed under aerobic conditions that juveniles transitioned from metabolism dominated by the Krebs cycle in early parenchymal stages, to aerobic acetate production dominating during later parenchymal stages, with malate dismutation being dominant in the bile duct stages [82]. Our data support the hypothesis that 21 day old juveniles *in vivo* are still utilising aerobic metabolism similar to *in vitro* maintained juveniles. Note that culturing *in vitro* juveniles under anaerobic conditions led to the death of juveniles suggesting that early developmental stages cannot rely on anaerobic metabolism for survival (S1 Fig).

Major components of both classical and neuropeptidergic signalling pathways in the liver fluke nervous system were downregulated *in vivo*, including synaptic vesicle cycle components and all elements of the neuropeptide processing pathway, indicating that these pathways are more prominent in the biology of *in vitro* maintained worms. Acetylcholine signalling was significantly upregulated in *in vitro* maintained juveniles. Notably, both acetylcholine and NPF/NPY neuropeptides have been identified as functioning in behaviours associated with nutrient acquisition and feeding in invertebrate species [83–86]. Acetylcholine signalling has been shown to function in the modulation of glucose transport in *S*. *mansoni* [84], whilst Drosophila NPF functions downstream of insulin signalling to regulate larval feeding [86]. The upregulation of these pathways may reflect the contrasting nutrient availabilities, resulting in changing behaviours of nutrient acquisition that could impact the growth and development of juveniles.

Of particular interest is the hypothesis that these neuronal signalling systems modulate growth and development of juvenile *F*. *hepatica* via mechanisms that influence stem cell dynamics and/or nutrient acquisition. Acetylcholine receptors have been found in all human stem cell populations, and in differentiated and undifferentiated cells types, supporting an important function beyond basic nervous system functioning and towards determination of cell fate and proliferation [87,88]. These receptors have also been identified as key players in cancer development due to their modulatory role in cell proliferation and expression in non-neuronal cell types [89]. The potential role of acetylcholine signalling in the growth and development of invertebrate species is unclear, although as early as 1985, the exogenous application of acetylcholine was reported to improve the growth and proliferation of *Drosophila* cell lines *in vitro* [90]. The activation of nicotinic acetylcholine receptors in *Caenorhabditis elegans* L2s resulted in delayed cell division and differentiation, stunting development [91]. Further, in situ hybridisation experiments showed extensive expression of nicotinic acetylcholine receptors in developing *Brugia malayi*, highlighting a specific role in embryogenesis and spermatogenesis [92]. NPF/NPY neuropeptides have also been linked to the modulation of regeneration and germline cell proliferation in invertebrates [93–96]. Non-neuronal NPF neuropeptides were shown to act as key regulators of mating-induced germline cell proliferation in *Drosophila* females [95]. In free living flatworms, NPF was found to accelerate pharyngeal regeneration in *Girardia tigrina* [93], whilst RNAi of prohormone convertase 2 (PC2) and non-neuronal NPY-8 had significant impacts on the reproductive maturation of *Schmidtea mediterranea*, suggesting neuropeptides are essential for germ cell differentiation [94]. It is therefore possible that differential expression of these neuronal signalling pathways relates to a fundamental role of the nervous system in regulating the growth and development of *F*. *hepatica* juveniles; the data would support the hypothesis that cell proliferation and/or related growth mechanisms are inhibited by a cadre of neuronal signalling molecules. It is also possible that an upregulation of neuronal signalling in *in vitro* juveniles may result from their maintenance in an unfamiliar environment lacking directional cues, heightening the expression of systems involved in host/niche finding behaviours.

miRNAs are an abundant class of regulatory genes that control many cellular and developmental processes [97]. Analysis of the miRNA complements of *in vitro* and *in vivo* maintained juvenile *F*. *hepatica* supports a significant role for these small RNAs in the modulation of developmental processes, including transcription and translation. The most significantly upregulated miRNA in *in vivo* juveniles was *fhe-let-7a-5p;* this miRNA is highly conserved across diverse organisms and has a significant role in the modulation of stem cells and the promotion of cell differentiation [98,99], consistent with its upregulation in the more highly developed *in vivo* maintained *F*. *hepatica* juveniles. *Fhe-mir-124-3p* plays a key role in neuronal cell differentiation [100] and is also thought to be a growth suppressor [101], potentially correlating with its higher expression in the smaller *in vitro F*. *hepatica* juveniles. The downregulation of *fhe-mir-124-3p* in *in vivo* juveniles may reflect the developmental stage of these juveniles with well-formed neuronal systems supported by comparatively lower levels of neuronal development. It was notable that GO term analysis of predicted miRNA targets largely correlated with the most significantly differentially expressed mechanisms identified through mRNA transcriptome analysis. In addition, Wnt signalling was identified as a process regulated by differentially expressed miRNAs leading to an overall downregulation of Wnt-associated mRNAs *in vivo*. miRNAs are well established regulators of Wnt signalling, a process essential for early organism development [102]. Downregulation of specific Wnt proteins, secreted frizzled-related proteins and frizzled GPCRs *in vivo*, suggest these proteins in particular have key roles in early stage developmental processes, consistent with later stage juveniles maintaining higher levels of tissue differentiation. Upregulation of low-density lipoprotein receptor-related protein (LRP) 5/6, a Wnt-associated gene, in *in vivo* juveniles suggests this protein is involved in later stage developmental processes. Although LRP5/6 is an essential co-receptor for canonical Wnt signalling [103], no other components of the canonical Wnt signalling pathway are upregulated *in vivo*, suggesting that either canonical Wnt signalling is not increased in later-stage juvenile development, or that LRP5/6 is a rate limiting component in Wnt-signalling pathway activity.

Overall, this study has provided unique insight into the biology of a helminth parasite maintained *in vitro* and encourages the exploitation of *in vitro* cultured parasites in functional genomics studies to inform aspects of *in vivo* biology and control target validation. Most of the transcriptomic differences between *in vitro* and *in vivo* maintained juvenile fluke relate to their divergent growth rate/stem cell proliferation and the developmental differences seen in the two groups of parasites. It should be noted that it is possible that the upregulation and/or downregulation of some genes may well encompass shifts in tissue type bias, however, where biology differs in relation to growth and development, new insights offer a basis for improving *in vitro* culture methods towards identifying key triggers for adult fluke development. The data expose a key role for miRNAs in coordinating the developmental differences seen in *in vivo* (fast growing) and *in vitro* (slow growing) juveniles. Further, the data highlight dramatic changes in the expression of neuronal signalling systems, consistent with a role for the nervous system in suppressing the growth and development of *F*. *hepatica* juveniles. The observations encourage the search for new potential control targets associated with signalling systems that regulate juvenile growth/development in liver fluke.

## Supporting information

S1 Data 21 day old juvenile *Fasciola hepatica* transcripts (MSTRG.1 > MSTRG.10300). (ZIP)

S2 Data 21 day old juvenile *Fasciola hepatica* transcripts (MSTRG.10301 > MSTRG.22211). (ZIP)

S1 Fig Survival of juvenile liver fluke in aerobic and anaerobic conditions. Percentage survival of juvenile liver fluke across 21 day period post excystment incubated under standard 5% CO_2_ conditions (red line) and in an anaerobic chamber (purple line). Juveniles showed significant and continuing higher rates of death when maintained in anaerobic chamber after 14 days (2-way ANOVA with Šídák’s multiple comparisons test; ****)
(PDF)

S2 Fig Components of N-glycan biosynthesis and processing pathways downregulated in *in vivo* maintained *F*. *hepatica* juveniles. Differential expression (log_2_FC) of protein glycosylating genes associated with N-glycan biosynthesis and processing. Genes identified in *F*. *hepatica* by McVeigh *et al*. [52] KEGG pathway analysis using R (v.3.6.2), gage (v.2.36.0) and pathview (v.1.26.0) packages identified a significant downregulation of N glycan biosynthesis in *in vivo* maintained juveniles (P≤0.05). Abbreviations; ALG N-glycan precursor synthesis- *Fh-*ALG(-3, -9) = dolichyl-P-Man:Man(5)GlcNAc(2)-PP-dolichol alpha-1,3-mannosyltransferase, *Fh-*ALG5 = dolichyl-phosphate beta-glucosyltransferase, *Fh-*ALG7 = UDP-N-acetylglucosamine—dolichyl-phosphate N-acetylglucosaminephosphotransferase; Oligosaccharyltransferase complex components- OST48 = dolichyl-diphosphooligosaccharide—protein glycosyltransferase subunit, *Fh-*RPN(-1, -2)&Fh-STT3(-A, -B) = dolichyl-diphosphooligosaccharide—protein glycosyltransferase subunit; N-glycan processing- *Fh*-GCNT2 = N-acetyllactosaminide beta-1,6-N-acetylglucosaminyl-transferase, *Fh-*FUT8 = alpha-(1,6)-fucosyltransferase, B4GALT = beta-1,4-galactosyltransferase, B4GALTNT = Beta—n-acetylgalactosaminyltransferase, EDEM1 = ER degradation-enhancing alpha-mannosidase-like protein, UGGT = UDP-glucose:glycoprotein glucosyltransferase, MAN2B1 = alpha-mannosidase.
(PDF)

S1 Table Transcriptome assembly and annotation statistics. (XLSX)

S2 Table Annotations for all 19,343 genes identified from transcriptomic analysis and annotated via BLAST analysis. (XLSX)

S3 Table Annotations for all differentially expressed genes from *in vitro* and *in vivo* transcriptome datasets annotated via BLAST analysis. (XLSX)

S4 Table A. Distribution of transcripts across *in vitro* and *in vivo* transcriptome replicates. B. Transcripts only present in *in vivo* and *in vitro* datasets.
(XLSX)

S5 Table Transcripts associated with neoblast proliferation. (XLSX)

S6 Table *Fasciola hepatica* cathepsin genes. (XLSX)

S7 Table Differentially expressed genes associated with aerobic and anaerobic carbohydrate metabolism. (XLSX)

S8 Table Genes associated with neuropeptide signalling system of *Fasciola hepatica*. (XLSX)

S9 Table A. miRNAs identified from liver fluke 21 day old juveniles maintained *in vitro* and *in vivo*. B. Differentially expressed miRNAs across maintenance treatments and predicted mRNA targets also differentially expressed
(XLSX)
