# Supplementary material for: Transcriptomic analysis supports a role for the nervous system in regulating growth and development of Fasciola hepatica juveniles
Source: PLoS Negl Trop Dis. 2022 Nov 7;16(11):e0010854. doi: 10.1371/journal.pntd.0010854 (PMC9639813; doi:10.1371/journal.pntd.0010854)
Supplement: S1 Fig — Percentage survival of juvenile liver fluke across 21 day period post excystment incubated under standard 5% CO2 conditions (red line) and in an anaerobic chamber (purple line). Juveniles showed significant and continuing higher rates of death when maintained in anaerobic chamber after 14 days (2-way ANOVA with Šídák’s multiple comparisons test; ****) (PDF) [file pntd.0010854.s003.pdf]

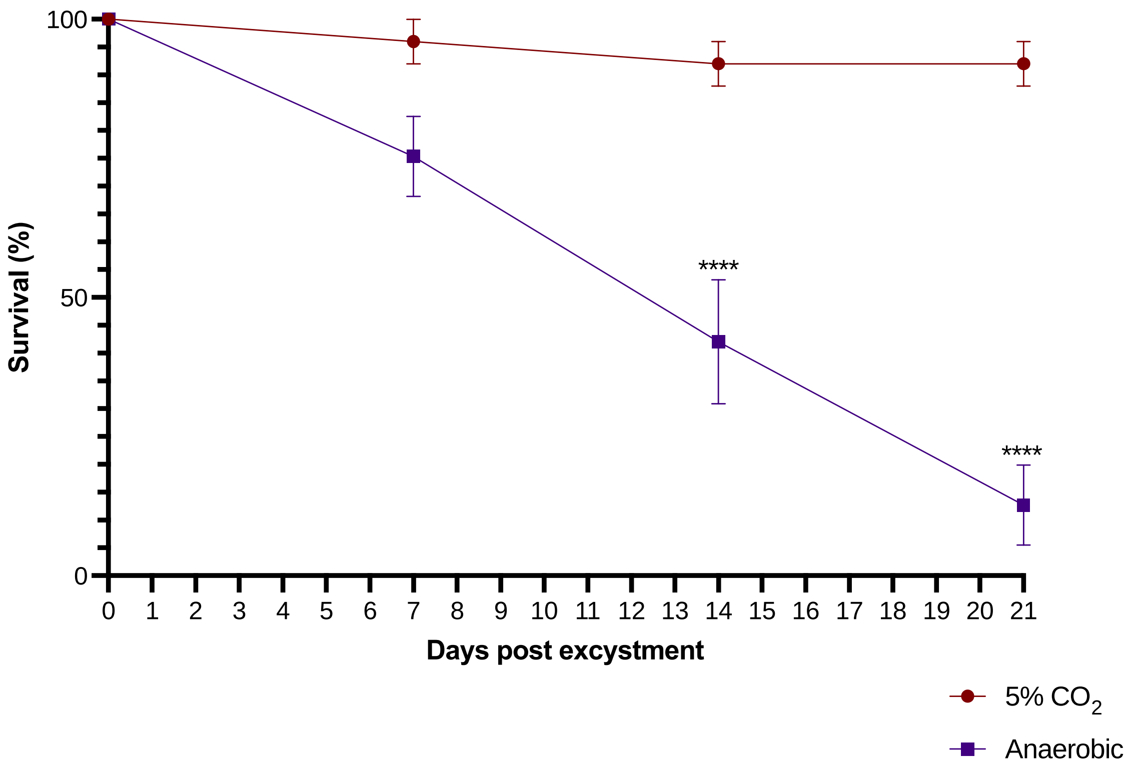

S1 Figure. Survival of juvenile liver fluke in aerobic and anaerobic conditions. Percentage survival of juvenile liver fluke across 21 day period post excystment incubated under standard 5% CO_2 conditions (red line) and in an anaerobic chamber (purple line). Juveniles showed significant and continuing higher rates of death when maintained in anaerobic chamber after 14 days (2-way ANOVA with Šídák's multiple comparisons test; ****
