# Supplementary material for: Transcriptomic analysis supports a role for the nervous system in regulating growth and development of Fasciola hepatica juveniles
Source: PLoS Negl Trop Dis. 2022 Nov 7;16(11):e0010854. doi: 10.1371/journal.pntd.0010854 (PMC9639813; doi:10.1371/journal.pntd.0010854)
Supplement: S2 Fig — Differential expression (log2FC) of protein glycosylating genes associated with N-glycan biosynthesis and processing. Genes identified in F. hepatica by McVeigh et al. [52] KEGG pathway analysis using R (v.3.6.2), gage (v.2.36.0) and pathview (v.1.26.0) packages identified a significant downregulation of N glycan biosynthesis in in vivo maintained juveniles (P≤0.05). Abbreviations; ALG N-glycan precursor synthesis- Fh-ALG(-3, -9) = dolichyl-P-Man:Man(5)GlcNAc(2)-PP-dolichol alpha-1,3-mannosyltransferase, Fh-ALG5 = dolichyl-phosphate beta-glucosyltransferase, Fh-ALG7 = UDP-N-acetylglucosamine—dolichyl-phosphate N-acetylglucosaminephosphotransferase; Oligosaccharyltransferase complex components- OST48 = dolichyl-diphosphooligosaccharide—protein glycosyltransferase subunit, Fh-RPN(-1, -2)&Fh-STT3(-A, -B) = dolichyl-diphosphooligosaccharide—protein glycosyltransferase subunit; N-glycan processing- Fh-GCNT2 = N-acetyllactosaminide beta-1,6-N-acetylglucosaminyl-transferase, Fh-FUT8 = alpha-(1,6)-fucosyltransferase, B4GALT = beta-1,4-galactosyltransferase, B4GALTNT = Beta—n-acetylgalactosaminyltransferase, EDEM1 = ER degradation-enhancing alpha-mannosidase-like protein, UGGT = UDP-glucose:glycoprotein glucosyltransferase, MAN2B1 = alpha-mannosidase. (PDF) [file pntd.0010854.s004.pdf]

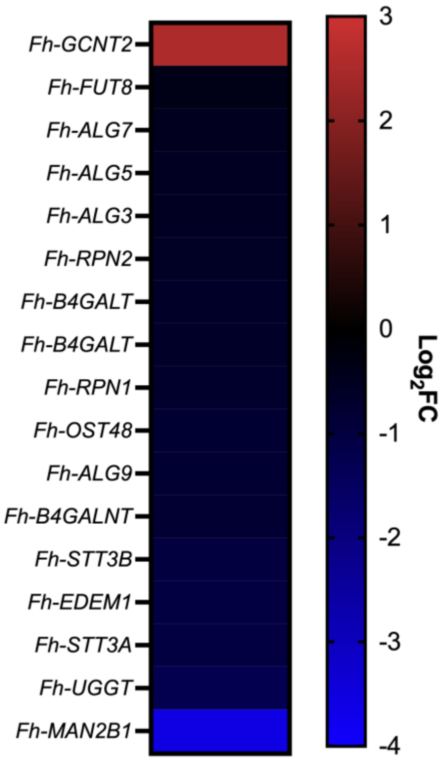

S2 Figure. Components of N-glycan biosynthesis and processing pathways downregulated in *in vivo* maintained *F. hepatica* juveniles. Differential expression (log_2FC) of protein glycosylating genes associated with N-glycan biosynthesis and processing. Genes identified in *F. hepatica* by McVeigh et al. [52] KEGG pathway analysis using R (v.3.6.2), gage (v.2.36.0) and pathview (v.1.26.0) packages identified a significant downregulation of N glycan biosynthesis in *in vivo* maintained juveniles ($P \leq 0.05$). Abbreviations; ALG N-glycan precursor synthesis- *Fh*-ALG(-3, -9)=dolichyl-P-Man:Man(5)GlcNAc(2)-PP-dolichol alpha-1,3-mannosyltransferase, *Fh*-ALG5=dolichyl-phosphate beta-glucosyltransferase, *Fh*-ALG7= UDP-N-acetylglucosamine--dolichyl-phosphate N-acetylglucosaminephosphotransferase; Oligosaccharyltransferase complex components- OST48= dolichyl-diphosphooligosaccharide--protein glycosyltransferase subunit, *Fh*-RPN(-1, -2)&Fh-STT3(-A, -B)= dolichyl-diphosphooligosaccharide--protein glycosyltransferase subunit; N-glycan processing- *Fh*-GCNT2=N-acetyllactosaminide beta-1,6-N-acetylglucosaminyl-transferase, *Fh*-FUT8=alpha-(1,6)-fucosyltransferase, B4GALT=beta-1,4-galactosyltransferase, B4GALTNT=Beta--n-acetylgalactosaminyltransferase, EDEM1=ER degradation-enhancing alpha-mannosidase-like protein, UGGT= UDP-glucose:glycoprotein glucosyltransferase, MAN2B1=alpha-mannosidase.
